# Degradation of MinD oscillator complexes by *Escherichia coli* ClpXP

**DOI:** 10.1074/jbc.RA120.013866

**Published:** 2020-12-10

**Authors:** Christopher J. LaBreck, Catherine E. Trebino, Colby N. Ferreira, Josiah J. Morrison, Eric C. DiBiasio, Joseph Conti, Jodi L. Camberg

**Affiliations:** Department of Cell & Molecular Biology, The University of Rhode Island, Kingston, Rhode Island, USA

**Keywords:** cell division, min system, MinD, ClpXP, Z-ring, divisome, proteolysis, AAA+ ATPase, *Escherichia coli*, AAA+, ATPase Associated with diverse cellular Activities, EPSCoR, Established Program to Stimulate Competitive Research, MES, (N-morpholino)ethanesulfonic acid, SUVs, small unilamellar vesicles, TCEP, Tris(2-carboxyethyl)phosphine, TEM, transmission electron microscopy

## Abstract

MinD is a cell division ATPase in *Escherichia coli* that oscillates from pole to pole and regulates the spatial position of the cell division machinery. Together with MinC and MinE, the Min system restricts assembly of the FtsZ-ring to midcell, oscillating between the opposite ends of the cell and preventing FtsZ-ring misassembly at the poles. Here, we show that the ATP-dependent bacterial proteasome complex ClpXP degrades MinD in reconstituted degradation reactions *in vitro* and *in vivo* through direct recognition of the MinD N-terminal region. MinD degradation is enhanced during stationary phase, suggesting that ClpXP regulates levels of MinD in cells that are not actively dividing. ClpXP is a major regulator of growth phase–dependent proteins, and these results suggest that MinD levels are also controlled during stationary phase. *In vitro*, MinC and MinD are known to coassemble into linear polymers; therefore, we monitored copolymers assembled *in vitro* after incubation with ClpXP and observed that ClpXP promotes rapid MinCD copolymer destabilization and direct MinD degradation by ClpXP. The N terminus of MinD, including residue Arg 3, which is near the ATP-binding site in sequence, is critical for degradation by ClpXP. Together, these results demonstrate that ClpXP degradation modifies conformational assemblies of MinD *in vitro* and depresses Min function *in vivo* during periods of reduced proliferation.

Cytokinesis in prokaryotes is a highly organized cellular process wherein a network of widely conserved cell division proteins function together to divide a single bacterial cell into two identical daughter cells (1). In *Escherichia coli*, cell division commences with the assembly of a large ring-like protein structure termed the Z-ring, which contains bundled polymers of the GTPase FtsZ, and FtsZ-interacting proteins including FtsA and ZipA, and serves as the site of constriction (2). The Z-ring is a highly dynamic structure wherein FtsZ subunits are rapidly exchanged with a cytoplasmic pool *via* cycles of GTP binding and hydrolysis (3). Many proteins interact with FtsZ to spatially and temporally regulate Z-ring assembly, and a number of these proteins modulate FtsZ dynamics in the Z-ring (4).

The Min system of *E. coli* functions to spatially regulate the site of cell division by inhibiting establishment of the Z-ring near the cell poles. MinD is one of three components of the Min system of proteins in *E. coli*, which includes MinC, MinD, and MinE. These proteins oscillate across the longitudinal axis of the cell to prevent Z-ring assembly at the poles in *E. coli* (5). The Min system is used by several taxa to regulate division-site selection; however, the oscillation observed in *E. coli* is not preserved across all organisms that contain a Min system, and some organisms lack a Min system entirely (5). MinC directly interacts with FtsZ to disrupt GTP-dependent polymerization *in vitro* (6, 7, 8). The cellular distribution of MinC is determined by MinD *via* a direct protein–protein interaction. MinD is a member of the Walker A cytoskeletal ATPases protein family and contains a deviant Walker A motif (9, 10). MinD associates with the cytoplasmic membrane in an ATP-bound dimer conformation *via* a C-terminal membrane targeting sequence. MinE binds to MinD, stimulating ATP hydrolysis and displacement of MinD from the membrane (11, 12).

MinC and MinD from several organisms, including *E. coli*, assemble into ATP-dependent cofilaments *in vitro* (13, 14, 15, 16). The Lowe group solved a crystal structure of the *Aquifex aeolicus* MinCD complex, which supports a model in which *A. aeolicus* copolymers contain alternating MinC and MinD dimers (13, 14). In *E. coli*, residues on the surface of the C-terminal domain of MinC are important for copolymerization with MinD (7, 13). Although several groups have reported copolymer formation *in vitro*, the physiological consequences of MinCD assembly *in vivo* are largely unknown. The Lutkenhaus group reported that MinD mutants that fail to polymerize with MinC, but still interact with MinC at the membrane, do not result in functional defects *in vivo* (17). Although copolymers are not essential to complete division *in vivo*, their assembly may modify Min patterning or oscillation rates *in vivo* through direct competition of accessible MinD surfaces by MinC and MinE (7).

In several prokaryotes, cytokinesis is regulated proteolytically by the two-component ATP-dependent protease ClpXP (18). In *E. coli*, targeted degradation of FtsZ by ClpXP modulates Z-ring dynamics during the division process (4, 19, 20). Additional *E. coli* cell division proteins have also been identified as ClpXP proteolysis substrates, including ZapC (21) and MinD, which was previously implicated as a substrate in a proteomics study performed under DNA damage conditions (22, 23). ClpXP contains both an unfoldase, ClpX, which is a hexameric ring-like AAA+ (ATPase Associated with diverse cellular Activities) ATPase, and the compartmentalized serine protease, ClpP, which is composed of two stacked heptameric rings (24). The ClpX unfoldase contains an N-terminal substrate-binding domain, also called the zinc-binding domain, that can undergo dimerization in solution and engages some substrates, including FtsZ and phage lambda O (λO) protein, in addition to substrate-specific adaptor proteins, such as SspB (20, 25, 26, 27). After engagement of a substrate, ClpX uses ATP hydrolysis to unfold and translocate substrates through its axial channel into the central chamber of ClpP for degradation (28, 29). Furthermore, ClpXP is a major regulator of protein stability and intracellular protein levels during stationary-phase adaptation and other stress conditions (23, 30).

Here, to determine if *E. coli* ClpXP regulates Min system function in *E. coli* by direct degradation of MinD and to understand how ClpX targets MinD, we reconstituted *in vitro* degradation assays with MinD and MinD-containing complexes, including MinCD copolymers. We show that ClpXP degrades MinD and destabilizes MinCD copolymers *in vitro*. Destabilization of MinCD copolymers is another example, in addition to FtsZ, which shows that ClpXP-dependent remodeling and degradation of large polymeric protein assemblies lead to their disassembly. We further demonstrate that ClpXP degrades MinD during stationary phase and that intracellular ClpXP levels modify Min function *in vivo*.

## Results

### ClpXP degrades MinD *in vitro*

**Figure 1:**
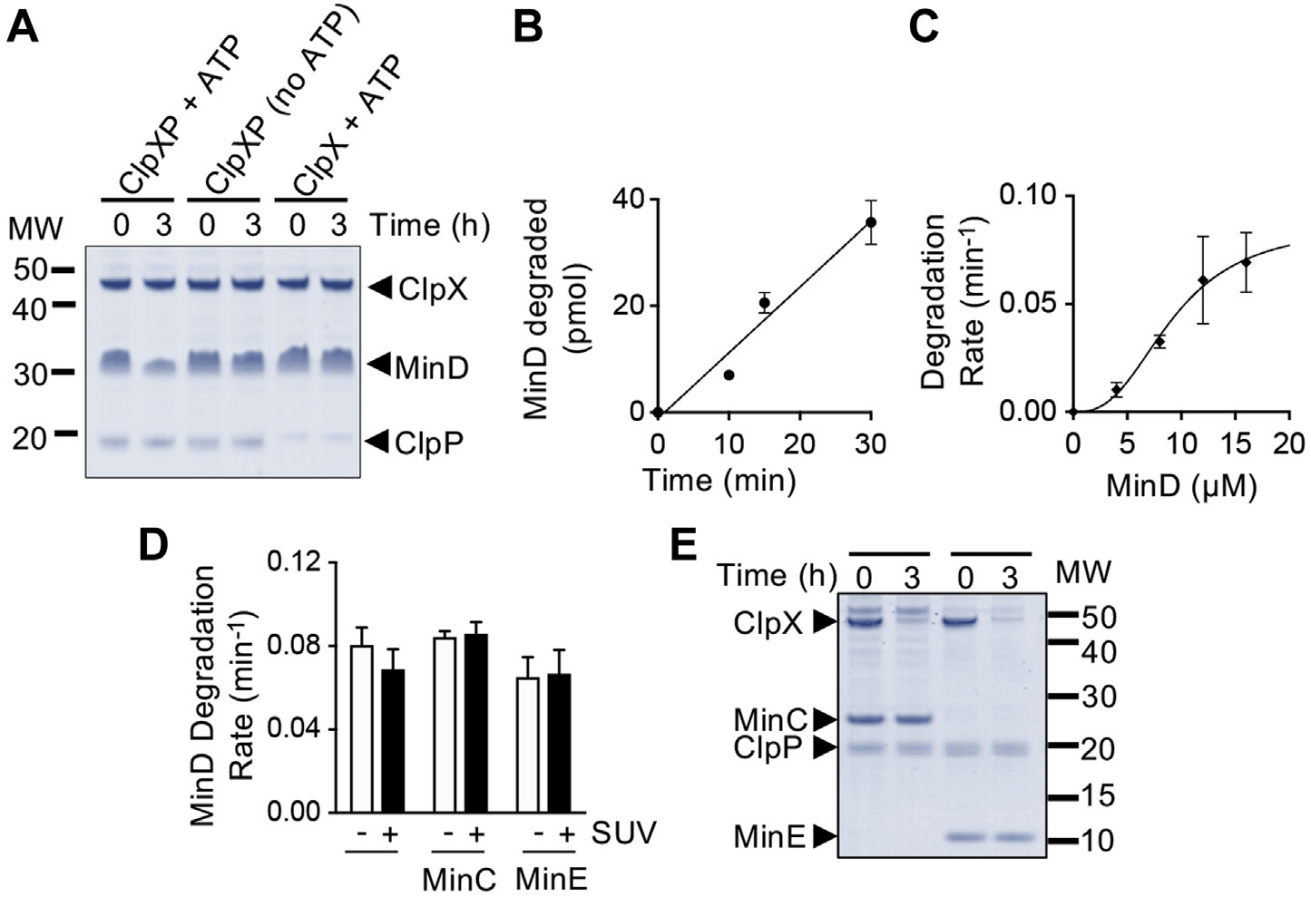
**ClpXP degrades MinD *in vitro*.***A,* MinD degradation was measured by monitoring the loss of MinD protein after 3 h in reactions containing MinD (6 μM), ClpX (1.0 μM), ClpP (1.2 μM), ATP (8 mM), and an ATP-regenerating system, where indicated. Samples collected at 0 and 3 h were analyzed by SDS–PAGE and Coomassie staining. *B,* degradation of fluorescent MinD was monitored in reactions containing ClpXP (0.8 μM), FL-MinD (10 μM), and ATP (8 mM) with a regenerating system for 30 min. Degradation products were collected by ultrafiltration and quantified by fluorescence. *C,* the rate of fluorescent MinD degradation was determined for reactions containing ClpXP (0.8 μM), FL-MinD (0–16 μM), and ATP (8 mM) and a regenerating system. *D,* MinD degradation was monitored in reactions containing ClpXP (0.8 μM), ATP (8 mM), and a regenerating system, with FL-MinD (10 μM), MinC (5 μM), MinE (10 μM), and SUVs (0.25 mg ml^−1^), where indicated. *E,* the amount of MinC or MinE after 0 and 3 h was visualized by SDS–PAGE in reactions containing MinC (6 μM), MinE (6 μM), where indicated, ClpX (1.0 μM), ClpP (1.2 μM), ATP (8 mM), and a regenerating system, as described in Experimental procedures. In *B* through *D*, data from at least three replicates are shown as mean ± SEM. MW, molecular weight; SUV, small unilamellar vesicle.

MinD was previously identified as a substrate for ClpXP degradation in *E. coli* W3110 (22). *In vivo*, deletion of either *clpX* or *clpP* from a *minC* deletion strain in *E. coli* MG1655 leads to a synthetic filamentous phenotype during exponential growth (31). FtsZ, the major component of the Z-ring, is also degraded by ClpXP, and an imbalance of FtsZ levels leads to filamentation (4, 20). To further understand how degradation of MinD by ClpXP impacts Min function during division, we first developed an *in vitro* degradation assay using purified proteins. MinD (6 μM) was incubated with ClpX (1.0 μM), ClpP (1.2 μM), and ATP for 3 h, and degradation was measured by monitoring the loss of full-length MinD in the reaction with time (Fig. 1*A*). After incubation of MinD with ClpXP and ATP, we detected 45.9% less MinD after 3 h (Fig. 1*A*); however, when either ATP or ClpP was omitted from reactions, the level of MinD did not change over the course of the experiment indicating that MinD is degraded by ClpXP in an ATP-dependent manner (Fig. 1*A*).

To quantitatively measure the rate of degradation in reconstituted reactions *in vitro*, we labeled MinD with Alexa fluor 488 and measured degradation by monitoring fluorescent peptides released following incubation with ClpXP and ATP. Degradation reactions containing ClpXP (0.8 μM), ATP (8 mM), and MinD (10 μM) were stopped by the addition of EDTA (50 mM), and fluorescent peptides were collected by ultrafiltration and quantified by fluorescence. We observed that the amount of MinD degraded increased linearly over the course of 30 min (Fig. 1*B*). Next, we examined the rate of MinD degradation by ClpXP with increasing MinD concentration (0–16 μM). We observed a concentration-dependent increase in the rate of MinD degradation, which plateaus near 20 μM MinD, with a rate of 0.08 ± 0.01 min^−1^ (Fig. 1*C*). The degradation rate of another substrate, FtsZ, has also been shown to increase with increasing substrate concentration, which suggests a low-affinity interaction at low substrate concentrations (4, 20). Together, these results demonstrate that ClpXP recognizes and degrades MinD in a concentration-dependent manner.

In the presence of ATP, MinD binds to *E. coli* phospholipids by inserting a C-terminal amphipathic helix into the phospholipid bilayer and recruits MinC to phospholipids *in vitro*. MinE also binds to membrane-associated MinD *in vitro*. MinE stimulates ATP hydrolysis by MinD in the presence of phospholipids, and then MinD dissociates from the phospholipid bilayer (32, 33). Because MinD is capable of binding directly to MinC, MinE, and phospholipids, we tested if the addition of *E. coli* phospholipids, prepared as small unilamellar vesicles (SUVs), modifies the rate of MinD degradation in the absence and the presence of MinC and MinE. We observed that during the interaction with SUVs, MinC (5 μM) or MinE (10 μM) had no significant impact on the rate of MinD degradation by ClpXP under the conditions tested (Fig. 1*D*). Finally, to confirm that ClpXP does not also degrade MinC or MinE, we monitored degradation of both MinC and MinE, but detected no proteolysis of either protein after 3 h, and autoproteolysis of ClpX was observed (Fig. 1*E*).

### ClpXP antagonizes MinCD copolymers

**Figure 2:**
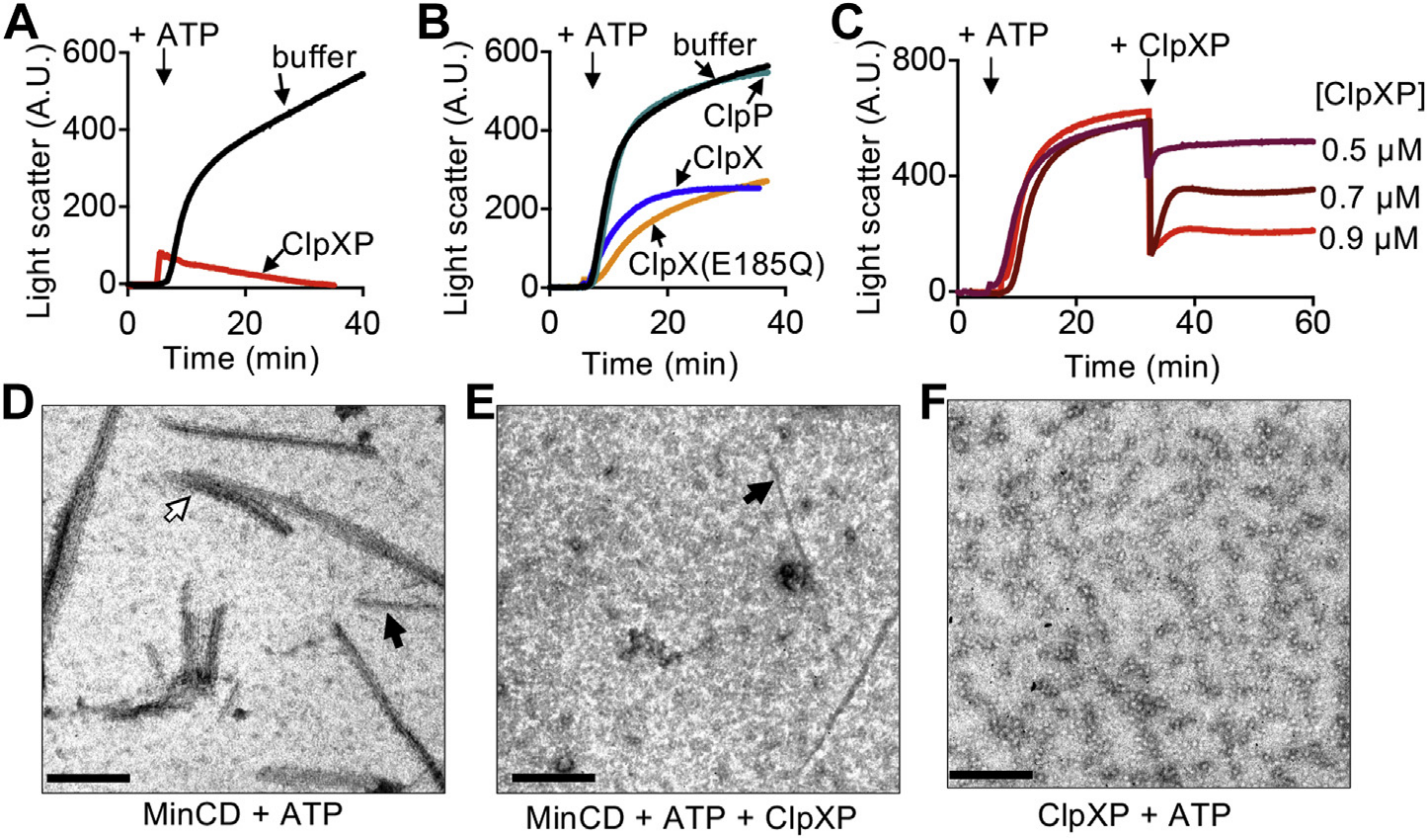
**ClpXP prevents MinCD copolymer assembly.***A,* MinCD copolymer assembly was monitored by 90° light scatter in reactions containing MinC (4 μM), MinD (8 μM), ClpX (0.75 μM), and ClpP (0.9 μM), where indicated. After 5 min, ATP (8 mM) was added, and reactions were monitored for an additional 30 min. *B,* MinCD copolymer assembly was monitored by light scatter as described in (*A*) in reactions containing MinC (4 μM), MinD (8 μM), with either ClpX (0.75 μM), ClpX(E185Q) (0.75 μM), ClpP (0.9 μM), or equivalent buffer, where indicated. Data shown are representative of at least three replicates. *C,* MinCD copolymers were assembled with MinC (4 μM), MinD (8 μM), and ATP (8 mM) and monitored by 90° light scatter. After 30 min, where indicated, ClpXP (0.5–0.9 μM) was added. Light scatter was monitored for an additional 30 min. In (*A*), (*B*), and (*C*), curves are representative of at least three replicates. *D,* polymers were visualized by TEM in reactions containing MinC (4 μM), MinD (8 μM), and ATP (8 mM) alone and (*E*) with ClpXP (0.75 μM). *F,* ClpXP (0.75 μM) and ATP complexes were visualized as described in (*E*). Reactions for TEM were applied to carbon-coated grids, fixed with glutaraldehyde, and stained with uranyl acetate. In *D* and *E*, *black arrows* indicate examples of single-stranded copolymers and *white arrow* indicates an example of copolymer bundles. The scale bar represents 200 nm.

MinC and MinD from *E. coli, Pseudomonas aeruginosa*, and *A. aeolicus* readily form copolymers in the presence of ATP composed of alternating dimers (13, 14, 15, 16). ClpXP is known to disassemble FtsZ polymers *in vitro* (4, 20); therefore, we tested if ClpXP could also prevent assembly of alternating MinCD copolymers or destabilize them after they assemble. First, to test if the presence of ClpXP in a MinCD assembly reaction reduces or prevents copolymerization of MinD with ATP and MinC *in vitro*, we monitored 90° light scatter of reactions containing MinD (8 μM), MinC (4 μM), and then added ATP alone or with ClpX and/or ClpP, where indicated (Fig. 2, *A*–*B*). Light scatter was then monitored for an additional 30 min. The addition of ATP without ClpXP stimulated robust copolymer formation; however, the addition of ATP and ClpXP lead to a small increase in light scatter that rapidly decreased with time (Fig. 2*A*). Next, we tested if ClpX impairs MinCD assembly without ClpP because inhibition of MinCD copolymer assembly could potentially result from MinD unfolding by ClpX, but not degradation. Interestingly, we found that the addition of ClpX resulted in a 45% inhibition of copolymerization (Fig. 2*B*). To determine if the inhibition required ATP hydrolysis, and therefore also ATP-dependent substrate unfolding, or binding only, we tested if the ClpX ATPase mutant protein, ClpX(E185Q), which hexamerizes and binds substrates but does not unfold them, impairs copolymer formation. Similar to wildtype ClpX, we observed a 45% reduction in the light scatter increase in response to ATP addition, suggesting that the binding, but not unfolding, partially reduces MinCD copolymer abundance (Fig. 2*B*). Together, these results suggest that ClpX prevents MinCD assembly independently of ClpP and ATP hydrolysis, but that ClpXP is substantially more effective for preventing assembly and/or destabilizing MinCD copolymers (Fig. 2, *A*–*B*). Addition of an equivalent volume of buffer or ClpP alone does not inhibit copolymer formation in control experiments (Fig. 2*B*). Together, our results suggest that MinCD copolymer assembly is prevented by ClpXP *in vitro* and, to a lesser extent, by ClpX.

**Figure 3:**
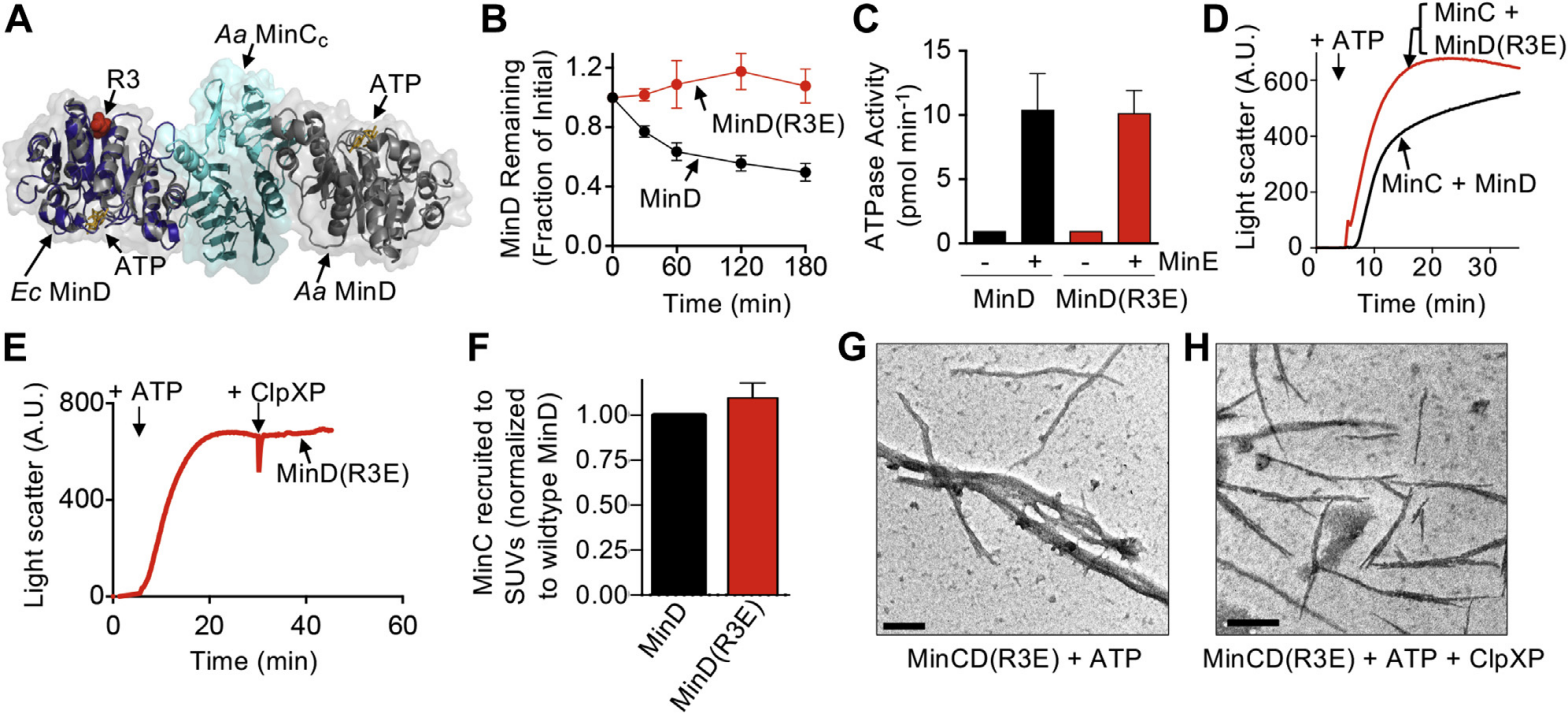
**MinD(R3E) is defective for degradation by ClpXP.***A,* Arg 3 of MinD is located near the ATP-binding site (G10 through T17) in the MinD sequence. *Escherichia coli* MinD (*blue*) was modeled onto a single protomer of *Aquifex aeolicus* MinD (*gray*) in complex with MinC C-domain dimer (*cyan*) (protein data bank: 4V02) (13). *B,* degradation was monitored in reactions containing ClpX (0.75 μM), ClpP (0.9 μM), ATP (8 mM), an ATP-regenerating system, and MinD (6 μM) or MinD(R3E) (6 μM), where indicated. *C,* ATP hydrolysis by MinD(R3E) with and without stimulation by MinE and SUVs was measured by monitoring phosphate release over 10 min in reactions containing SUVs (1 mg ml^−1^), ATP (4 mM), and MinD (8 μM), MinD(R3E) (8 μM), and MinE (16 μM), where indicated. *D,* copolymer formation by MinD and MinD(R3E) with MinC was compared by monitoring 90° light scatter as described in Experimental procedures. Reactions contained MinC (4 μM), ATP (4 mM), and MinD (8 μM) or MinD(R3E) (8 μM), where indicated. Light scatter was monitored for 5 min, ATP was added, and monitored for an additional 30 min. Each curve is representative of at least three replicates. *E,* copolymer formation by MinD(R3E) with MinC was compared by monitoring 90° light scatter with ATP for 30 min, and then ClpXP was added to the reaction, and light scatter was monitored for an additional 30 min as described in Experimental procedures. *F,* the ability of MinD(R3E) to recruit MinC to SUVs was measured and compared with MinD. Reactions contained SUVs (0.25 mg ml^−1^), ATP (4 mM), MinC (4 μM), MinD (4 μM), or MinD(R3E) (4 μM), as described in Experimental procedures. *G,* polymers containing MinC and MinD(R3E) were visualized by TEM in reactions containing MinC (4 μM), MinD(R3E) (8 μM), and ATP (8 mM) alone and (*H*) with ClpXP (0.8 μM), where indicated. The scale bar represents 200 nm. SUVs, small unilamellar vesicles.

Next to determine if ClpXP destabilizes preassembled MinCD copolymers, we performed an order of addition experiment. First, copolymers were preassembled with ATP, and then ClpXP (0.5–0.9 μM) was added, and we monitored light scatter for another 30 min to detect MinCD copolymers. After assembly of MinCD polymers, addition of ClpXP to the reaction led to a rapid decrease in light scatter that correlated with increased ClpXP concentration (Fig. 2*C*). In contrast, the addition of buffer, ClpX, or ClpP failed to destabilize assembled MinCD copolymers (Fig. S1*A*). Finally, we directly visualized copolymers *via* negative staining transmission electron microscopy (TEM) and compared MinCD copolymer abundance and morphology to copolymers incubated with ClpXP. Consistent with previous reports, we observed MinCD copolymers with ATP (8 mM) (Fig. 2*D*). Many copolymers were straight and single stranded; however, we also observed bundles of copolymers. Next, reactions containing ClpXP (0.8 μM) with ATP (8 mM) alone or added to MinCD copolymers, preassembled with ATP, were incubated for 15 min, and reaction products were analyzed by TEM. In the presence of ClpXP, we observed fewer copolymers that were shorter and spread more sparsely across the grid (Fig. 2*E*), compared with copolymers without ClpXP (Fig. 2*D*). To confirm that polymers were not observed in reactions containing ClpXP alone, we visualized ClpXP (0.9 μM) assembled with ATP (4 mM). We detected a homogeneous population of ClpXP particles (Fig. 2*F*) and did not observe any polymeric structures. These results suggest that the ClpXP destabilizes MinCD copolymers, and ClpX is sufficient to prevent assembly of MinCD copolymers through binding and is independent of ATP hydrolysis. In the structural model of MinCD, copolymers are comprised of alternating MinC and MinD dimers (13) (Fig. 3*A*). Therefore, it is possible that copolymer destabilization by ClpXP could arise from degradation of MinC or a failure of MinC to dimerize and/or interact with MinD. As described, we observed no degradation of MinC by SDS–PAGE after incubation with ClpXP and ATP (Fig. 1*E*); therefore, copolymer disassembly does not likely occur *via* MinC engagement. In a control experiment, Gfp–ssrA was rapidly degraded by ClpXP (Fig. S1*B*).

### The MinD N-terminal sequence contains residues that are important for degradation by ClpXP

Substrate recognition by ClpX is mediated by the presence of different sequence motifs, or degrons, at the N- or C-terminal regions of protein substrates (23, 34). The N terminus of MinD contains amino acids that bear similarity to the N motif-2 consensus motif (M-b-ϕ-ϕ-ϕ-X_5_-ϕ) identified for ClpX (22, 23) (^1^MARIIV-X_5_-G^12^). In the structural model of MinD, Arg 3 is present near the N terminus, outside the dimer interface, and accessible to the surface (Fig. 3*A*). To determine if this arginine is important for recognition by ClpX, we mutagenized the residue to glutamate and purified MinD(R3E). In degradation reactions with ClpXP *in vitro*, we observed that 50% of wildtype MinD was degraded in the first 60 min; however, we detected no MinD(R3E) degradation during the experiment under the conditions tested (Fig. 3*B*). To confirm that MinD(R3E) is not defective for function, we measured the ability of MinE to stimulate ATP hydrolysis of MinD(R3E) in the presence of SUVs. We observed that the ATP hydrolysis rate of MinD(R3E) (8 μM) was stimulated 10-fold by MinE (16 μM) and SUVs (1 mg ml^−1^), similar to wildtype MinD, suggesting that the amino acid substitution does not impair MinD function (Fig. 3*C*).

MinD(R3E) is defective for degradation by ClpXP but copolymerizes with MinC (Fig. 3*D*); therefore, we tested if whether copolymers containing MinD(R3E) and MinC are resistant to destabilization by ClpXP. As expected, we observed that ClpXP failed to destabilize preassembled MinC/MinD(R3E) polymers, in contrast to copolymers formed with MinC (4 μM) and wildtype MinD (8 μM) (Figs. 2*C* and 3*E*). Our results suggest that ClpXP destabilizes MinCD copolymers and that Arg 3 of MinD is important. Furthermore, MinD(R3E) (4 μM) recruited similar levels of MinC (4 μM) to SUVs compared with MinD (4 μM) (Fig. 3*F*) suggesting that it is not defective for known interactions *in vitro*. Finally, we visualized MinD(R3E)-containing copolymers assembled with MinD(R3E) (8 μM), MinC (4 μM), and ATP by TEM that were incubated with and without ClpXP (0.8 μM). As expected, copolymers were detected under both conditions, consistent with a failure of ClpXP to destabilize copolymers containing MinD(R3E) and MinC (Fig. 3, *G* and *H*).

### MinD degradation during stationary phase

**Figure 4:**
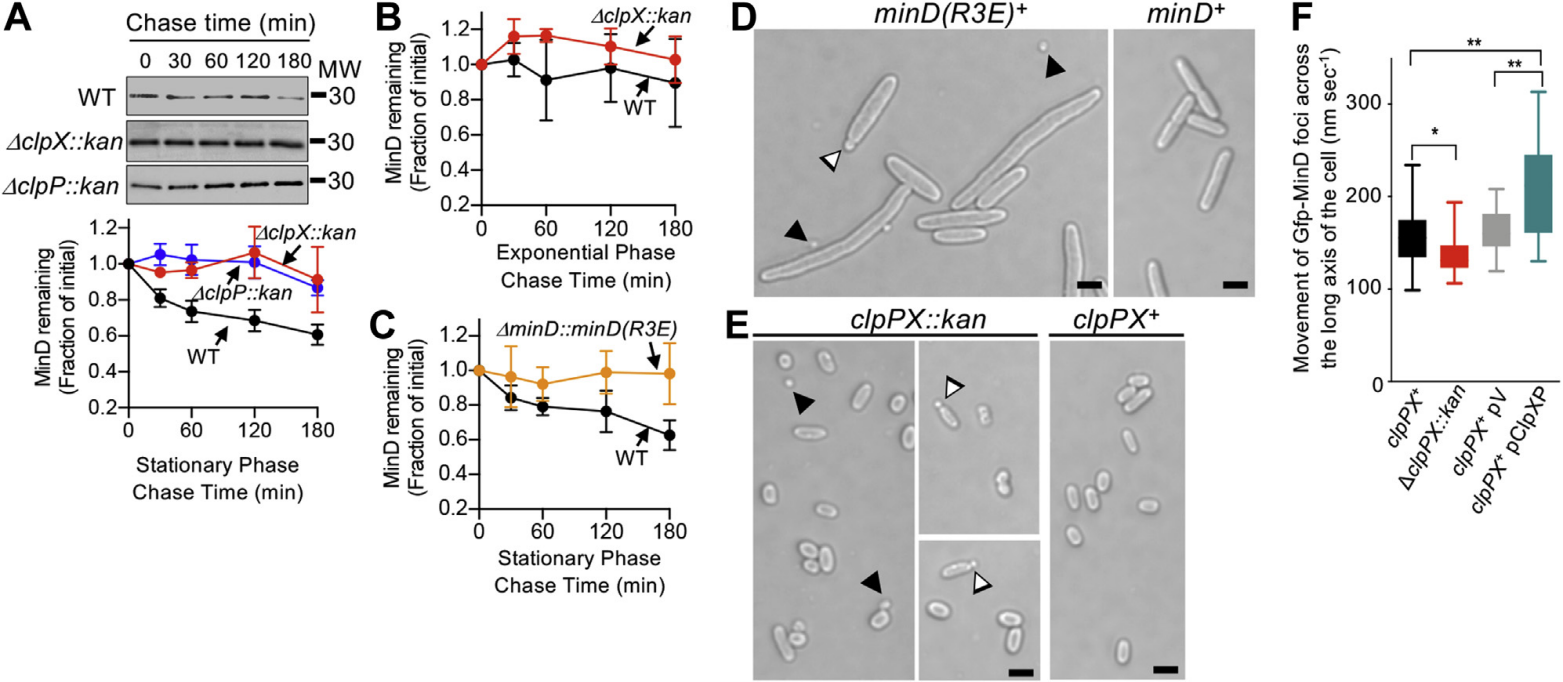
**MinD degradation *in vivo* by ClpXP.***A,* relative MinD levels were monitored in cells that were grown to stationary phase in Luria–Bertani (LB) and then treated with spectinomycin (200 μg ml^−1^). Cell extracts from wildtype MG1655, *clpX* (JC0259), and *clpP* (JC0263) deletion strains were analyzed by immunoblotting for MinD followed by densitometry. *B,* relative MinD levels were monitored with time in cells that were grown to log phase (absorbance of 0.3 AU at 600 nm) in LB and then treated with spectinomycin (200 μg ml^−1^). Cell extracts from wildtype MG1655 and *clpX* (JC0259) deletion strains were analyzed by immunoblotting for MinD followed by densitometry. *C,* relative levels of MinD(R3E) (ED0800) and MinD (MG1655), where indicated, were monitored with time in cells that were grown to stationary phase (16 h) in LB and then treated with spectinomycin (200 μg ml^−1^). Cell extracts were analyzed by immunoblotting with anti-MinD antibodies followed by densitometry. *D,* MG1655 cells containing *minD* or *minD(R3E)* (ED0800) grown in LB to log phase and visualized by differential interference contrast microscopy. *E,* MG1655 cells containing *clpPX* or deleted for *clpPX*, referred to as *clpPX::kan* (JC0208) grown in LB to stationary phase (19 h) and visualized by differential interference contrast microscopy. In *D* and *E*, *black arrows* indicate examples of minicells, and *white arrows* indicate examples of polar buds. The size bars represent 2 μm. *F,* cells expressing Gfp–MinD from the native locus were visualized by fluorescence microscopy to monitor polar oscillation of Gfp–MinD of log phase cultures. Oscillation rates of Gfp–MinD were measured in at least 20 cells expressing ClpXP from the chromosome (*clpPX*^*+*^*)* (CF005), deleted for *clpPX* (Δ*clpPX*::*kan*) (CF015), or overexpressing ClpXP from a vector (pClpXP) and compared with cells containing control vector pBr322 (pV) as described in Experimental procedures (*p* values are as follows, ∗ ≤ 0.01, ∗∗ < 0.005). MW, molecular weight; WT, wildtype.

To monitor protein turnover of MinD in the K-12 strain MG1655 and determine if ClpXP actively degrades MinD *in vivo* as well as *in vitro*, we performed an antibiotic chase experiment using spectinomycin to halt new protein synthesis and then monitored MinD levels by immunoblot. We observed that MinD is degraded in MG1655 cells from 16 h cultures (absorbance of 2.0 AU at 600 nm) with a long half-life of approximately 180 min (Fig. 4*A*). However, deletion of *clpX* or *clpP* prevents the observed degradation and significantly stabilizes the protein over the course of the experiment, indicating that ClpXP is predominantly responsible for the observed turnover in this strain under the conditions tested, and that other proteases, including ClpAP and Lon, likely do not extensively contribute to MinD turnover during stationary phase (Fig. 4*A*). We expect that MinD function occurs largely as cells divide during log phase growth; therefore, we compared MinD protein turnover in cultures of cells grown to an absorbance of 0.3 AU at 600 nm. We observed even slower MinD protein turnover relative to stationary phase, with MinD persisting for the duration of the chase and much longer than 180 min (Fig. 4*B*). MinD was similarly stable in log phase cells deleted for *clpX* (Fig 4*B*). Finally, to confirm that MinD protein turnover during stationary phase occurred through recognition of the MinD N-terminal region, we replaced *minD* at the native locus in the chromosome with a gene encoding the modified MinD mutant protein MinD(R3E), which is defective for ClpXP degradation (Fig. 3*B*). In antibiotic chase experiments, we observed that MinD(R3E) is not degraded during stationary phase, in contrast to wildtype MinD (Fig. 4*C*). To detect if cells expressing MinD(R3E) displayed cell division abnormalities, we measured cell lengths of MG1655 *minD::minD(R3E)* cells during log phase growth. We measured the average cell length of cells containing *minD(R3E)* in log phase to be 5.6 ± 0.4 μm (n = 145), which is 47% longer than cells containing wildtype *minD* (3.8 ± 0.2 μm [n = 200]). Moreover, 4 of 145 cells (2.8%) containing *minD(R3E)* were smaller than 1 μm in length, consistent with minicells, and polar buds were detected; no cells containing wildtype *minD* were smaller than 1 μm in length (Fig. 4*D*).

Intracellular MinD protein levels during log phase or stationary phase would be controlled by a balance of synthesis and degradation, with partitioning of Min proteins into daughter cells during division events. Therefore, we next tested if Min oscillations are more active or detectable in log phase versus stationary phase. To monitor MinD oscillation *in vivo*, we replaced *minD* in the chromosomal *min* operon with *gfp–minD* (Table 1)*.* Under the conditions tested, Gfp–MinD appears fully functional for regulating division in cells. Cells expressing Gfp–MinD have a mean cell length of 1.92 ± 0.04 μm (n = 200), which is similar to wildtype cells with a mean cell length of 1.93 ± 0.03 μm (n = 200), and no minicells were observed. We monitored productive oscillations in cells expressing Gfp–MinD and found that over half of the cells (51%) harvested from log phase cultures (absorbance of 0.3 AU at 600 nm) showed productive Gfp–MinD oscillations, whereas 38% of cells harvested from stationary phase 16 h cultures (absorbance of 2.0 AU at 600 nm) showed productive oscillations with weak overall fluorescence. These results suggest that the Min system is depressed in cells that are in stationary phase, which is not surprising because fewer cells should be actively dividing. We previously detected that MinD degradation by ClpXP observed *in vivo* in asynchronous cell populations occurs during stationary phase. Accordingly, we monitored Gfp–MinD oscillations in stationary phase cells deleted for *clpP* and *clpX* and detected an increase in the overall number of apparent oscillations detected during stationary phase to 45% of the total population.

**Table 1:** *E. coli* strains and plasmids

Strain or plasmid	Genotype	Source, reference, or construction
Strains		
MG1655	*LAM-rph-1*	(35)
BL21 (λDE3)	*F–ompT gal dcm lon hsdSB(rB- mB-) λ(DE3[lacI lacUV5-T7 gene 1 ind1 sam7 nin5])*	EMD Millipore, United States
CL0030	MG1655 Δ*minC*::*kan-Prha-parE*	(7)
CL0428	MG1655 Δ*minC::Pbad-gfp-minC*	(7)
CF020	MG1655 Δ*minC::Pbad-gfp-minC* Δ*clpPX::kan-Prha-parE*	CL0428; λred
CL0476	MG1655 Δ*minD::kan-Prha-parE*	MG1655; λred
CF005	MG1655 Δ*minD::gfp-minD*	CL0476; λred
CF015	MG1655 Δ*minD::gfp-minD* Δ*clpPX::kan-Prha-parE*	CF005; λred
ED0800	MG1655 Δ*minD::mind(R3E)*	CL0476; λred
JC0208	MG1655 Δ*clpPX::kan*	Lies & Maurizi^a^
JC0259	MG1655 Δ*clpX::kan*	(31)
JC0263	MG1655 Δ*clpP::kan*	(31)
Plasmids		
pEt-MinC	*kan*	(14)
pEt-MinD	*kan*	(14)
pEt-MinD(R3E)	*kan*	This study
pKD46	*amp*; λred recombinase	(36)
pKD267	*kan-Prha-parE*	(7)
pBAD33-*gfp*	*cat*	(7)
pBAD33-*gfp–minC*	*cat*	(7)
pBAD33-*gfp–minD*	*cat*	This study
pBR322	*amp*	(37)
pClpXP	*amp*	(20)

a This strain was provided by M. Lies and M. Maurizi.

If ClpXP contributes to cell length determination during stationary phase, then we would expect there to be a morphological defect in cells deleted for *clpPX*, and these cells would accumulate in culture and be apparent during stationary phase. Therefore, we examined the morphology of *clpPX* deletion cells during stationary phase growth and observed that 14 of 200 cells (7.5%) were smaller than 1 μm in length, which is consistent with minicells, and we detected the presence of polar buds (Fig. 4*E*). In contrast, only one cell expressing wildtype *clpX* and *clpP* was smaller than 1 μm in length (Fig. 4*E*). Together, our results suggest that ClpXP contributes to reduced Min function by degrading MinD in slow-growing cells, leading to fewer cells that exhibit Min oscillation during stationary phase.

Finally, we investigated if ClpXP degradation activity impacts or modifies Min patterning in cells exhibiting productive oscillations from log phase cultures. Therefore, we measured the rate of fluorescent foci movement from pole to pole by Gfp–MinD in cells with and without ClpXP (Fig. S2*A*). We observed polar oscillation of Gfp–MinD in live, dividing cells with movement of the fluorescent foci across the longitudinal axis of the cell at a rate of 160.4 ± 7.6 nm s^−1^ (Fig. 4*F*). In cells deleted for *clpP* and *clpX* (Table 1), denoted here as *clpPX*, we observed a modest but significant 15% reduction in the Gfp–MinD oscillation rate compared with cells with *clpPX* intact and controlling for similar cell length (Fig. 4*F*). The rate of oscillation by Gfp–MinD measured here is within error of the oscillation rate reported previously for Gfp–MinC in the chromosome, and also measured here at 163.3 ± 4.8 nm s^−1^ (Fig. S2*B*) (7). Accordingly, deletion of *clpPX* also led to a 15% reduced Gfp–MinC oscillation rate, which is dependent on MinD for oscillation (Fig. S2*B*). Surprisingly, overexpression of ClpXP from a vector that was previously shown to increase ClpX and ClpP levels by 4- and 75-fold, respectively, increased the oscillation rates of both Gfp–MinD and Gfp–MinC (Figs. 4*F* and S2*B*). Together, these results indicate that ClpXP degradation activity modulates MinC and MinD oscillation rates in dividing cells *in vivo*.

## Discussion

Here, we demonstrate that the two-component ATP-dependent protease ClpXP degrades the *E. coli* cell division protein MinD *in vitro* and confirm that MinD is also degraded by ClpXP *in vivo*, consistent with a previous report initially identifying MinD as a substrate (22); however, we observed that MinD is more susceptible to degradation by ClpXP during stationary phase than log phase suggesting that it is a growth phase–regulated protein. The N-terminal region of MinD is important for degradation and has a sequence similar to other substrates with predicted N-terminal degrons (23). We identified a specific residue in the MinD N-terminal region, Arg 3, that is important for recognition and degradation by ClpXP, and mutagenesis of Arg 3 does not impact MinD function *in vitro* (Fig. 3, *C*, *D*, and *F*). Although ClpX functions as a part of a proteasome complex with ClpP to regulate overall protein turnover of many substrates, such as RpoS *in vivo*, ClpX can also remodel protein substrates in the absence of ClpP. For example, ClpX remodels MuA transpososomes to regulate phage transposition and can function as a protein disaggregase (38, 39, 40). Given that ClpX is capable of remodeling protein complexes with and without ClpP, we investigated the ability of ClpX and ClpXP to modulate MinCD copolymer assembly *in vitro*. We found that ATP-driven coassembly of MinCD was partially prevented in the presence of ClpX and abolished in the presence of ClpXP (Fig. 2, *A*–*B*). Furthermore, copolymer formation was similarly impaired by ClpX and ClpX(E185Q), which contains a mutation in the Walker B motif and is defective for ATP hydrolysis, but still oligomerizes and binds to substrates (Fig. 2*B*) (41). Thus, ClpX alone antagonizes MinCD copolymer assembly *via* an ATP hydrolysis–independent holding mechanism. Consistent with these results, previous studies demonstrated that in *Bacillus subtilis*, ClpX impairs FtsZ polymer assembly through an ATP-independent mechanism (42, 43). ATP hydrolysis by ClpX is required for substrate unfolding and translocation but not substrate binding. Therefore, our results are consistent with ClpX inhibiting copolymerization *via* a binding/holding mechanism, where the direct interaction, without unfolding, may be sufficient to impair assembly or potentially cap copolymers.

In *E. coli*, ClpXP and ClpAP play critical roles in stationary phase adaptation and control of protein levels in nonproliferating cells that may be encountering nutrient deprivation or environmental stress (30). Moreover, ClpP levels are induced on transition into stationary phase, whereas ClpX levels remain largely constant (44). In addition, stationary phase or stressed cells may accumulate aggregates, and clearance of aggregates is important for mitigating their cytotoxicity. ClpXP also degrades *E. coli* FtsZ, which assembles into linear polymers, during division (4, 20). Eukaryotic members of the AAA+ protein family, including spastin and katanin, are also capable of microtubule polymer disassembly (45). Thus, an additional major function of ClpXP may be to disassemble polymer networks or aggregates in the cytoplasm. MinD and MinC from several organisms form large linear filaments that are readily observed by electron microscopy (7, 13, 14, 15). Here we show that *E. coli* MinCD copolymers are also rapidly destabilized by ClpXP *in vitro* (Fig. 2*C*). To date, several multimerizing protein substrates, including FtsZ, MuA, DPS, and MinD, as well as intracellular misfolded aggregates (38), are remodeled, disassembled, and/or degraded by ClpXP.

Gfp–MinD oscillation occurs in the absence of MinC, and cells expressing MinD mutant proteins that are impaired for copolymerization with MinC do not have obvious cell division defects *in vivo* (17, 46). However, copolymerization with MinC may limit the available population of MinD that is activated *in vivo*. This may occur through sequestration in the cytoplasm or concentration on the membrane, thereby modifying oscillation. We observed that ClpXP, and to a lesser extent ClpX, directly alters copolymer assembly and abundance *in vitro* (Figs. 2 and 3*E*, *G*, and *H*). Thus, ClpXP may also modulate the accessible population of MinD by modifying copolymer conformation. When we tracked total MinD protein turnover, we observed that MinD protein levels were relatively stable during log phase growth, and more susceptible to ClpXP proteolysis during stationary phase, although we obtained these results from asynchronous cell populations. It is currently unclear if the stationary phase MinD proteolysis that we observed serves a general housekeeping function of ClpXP, or if ClpXP destabilizes and degrades MinD-containing complexes to affect divisome function or alleviate a burden of intracellular ATP utilization consumed by MinD oscillation. Another study reported that intracellular MinD levels *in vivo* are also reduced under DNA damaging conditions (22). Notably, that study reported a short MinD half-life *in vivo* in *E. coli* W3110 of 5 min (22), suggesting that the overall extent of MinD stabilization and proteolysis may also be significantly impacted by the genetic background of the strain as well as growth phase in culture.

Alterations to ClpXP expression levels also modify Min oscillation rates in dividing cells (Fig. 4*F*). This likely results from direct degradation of MinD and/or MinD complex disassembly. The ratio of MinE to MinD is important for accumulation of MinD on the membrane, wave velocity, release from the membrane *in vitro* (47, 48), and oscillation *in vivo* (49). Therefore, degradation of MinD in the cell would increase the MinE:MinD ratio, likely increasing the rate of oscillation, consistent with our observation. With sufficient MinD degradation, Min oscillation could break down entirely. This could explain why fewer cells exhibit oscillations during stationary phase. Finally, there could be an indirect effect on Min oscillation that occurs in cells with altered degradation of FtsZ, which is also degraded by ClpXP, leading to modified Z-ring dynamics (4, 20). There is much that remains to be understood about the spatiotemporal coordination of Z-ring assembly and Min patterning, as well as bulk effects of membrane-associated MinD and FtsZ polymer mass and dynamics on cell cycle timing, constriction, or septation, which may be further regulated by proteolysis. However, it is clear that ClpXP proteolysis fine tunes division machinery function in *E. coli via* degradation of cell division proteins, including FtsZ and ZapC (4, 21, 31), and may further control cell division protein function during different growth phases, as with MinD, or under thermal stress, as with FtsZ (38).

## Experimental procedures

### Strain construction

Strains and plasmids are described in Table 1. Strains deleted for *clpX*, *clpP*, or the *clpPX* operon, and strains expressing Gfp–MinD, Gfp–MinC, and MinD(R3E) from the native loci in the chromosome were constructed using Lambda Red recombination and gene replacement, where indicated, using negative selection by induction of *parE* by rhamnose, as described (7, 36).

### Protein expression and purification

MinC, MinD, MinE, FtsZ, ClpX, ClpX(E185Q), and ClpP were each overexpressed from a plasmid in *E. coli* BL21 (λDE3) and purified as native proteins according to previously published methods (7, 14, 20, 50). MinD and ClpX mutant proteins were constructed by site-directed mutagenesis of plasmids containing *minD* or *clpX* using the QuickChange II XL Site-directed mutagenesis kit (Agilent) and confirmed by sequencing prior to purification. Gfp–ssrA was purified as described (51). Protein concentrations are reported as FtsZ monomers, Gfp–ssrA monomers, MinC dimers, MinD dimers, MinE dimers, ClpX hexamers, and ClpP tetradecamers.

MinD was labeled with Alexa fluor 488 (FL-MinD) according to manufacturer's protocol (Life Technologies). Briefly, dye Alexa fluor 488 C5-maleimide (0.2 mg) was added to MinD (2.5 mg) in buffer containing 50 mM Hepes, pH 7.0, 150 mM KCl, 10% glycerol, and 1 mM Tris(2-carboxyethyl)phosphine (TCEP) and incubated for 1 h at 23 °C. Reactions were quenched with β-mercaptoethanol, and unconjugated dye was removed by buffer exchange on a PD-10 column. The degree of labeling is 0.4 mol dye per mol of MinD. Labeled MinD function was confirmed to be similar to wildtype MinD in ATP hydrolysis assays, phospholipid-binding assays, and ATP-dependent copolymer assays with MinC.

### Phospholipid recruitment assays with MinC and MinD

MinD (4 μM) or MinD(R3E) (4 μM), where indicated, were added to reactions containing SUVs (0.25 mg ml^−1^), MinC (4 μM), and ATP (4 mM) in assembly buffer containing 50 mM of 2-(*N*-morpholino)ethanesulfonic acid (MES) with a pH of 6.5, 100 mM KCl, and 10 mM MgCl_2_. SUVs from *E. coli* extracts (Avanti Polar lipids) were prepared as described (52). Following the addition of ATP, reactions were incubated for 10 min at 30 °C, and SUV-containing protein complexes were collected by centrifugation at 14,000 × *g* at 23 °C, resuspended in lithium dodecyl sulfate loading buffer, and analyzed by SDS–PAGE and densitometry.

### ATP hydrolysis assays with MinD

ATP hydrolysis was measured by monitoring the amount of inorganic phosphate released after 10 min in reactions containing Tris buffer (20 mM; pH 7.5), KCl (50 mM), MgCl_2_ (10 mM), ATP (4 mM), MinD (8 μM), MinD(R3E) (8 μM), MinE (16 μM), and SUVs (1 mg ml^−1^), where indicated. Phosphate released in reactions was detected using Biomol green (Enzo Life Sciences) and compared with a phosphate standard curve.

### Degradation assays

FL-MinD (10 μM), or FL-MinD (4–16 μM), where indicated, was added to reactions containing ClpX (0.7 μM), ClpP (0.8 μM), MinC (5 μM), MinE (10 μM), and ATP (8 mM) with an ATP-regenerating system containing creatine phosphokinase (50 μg ml^−1^) and creatine phosphate (30 mM), where indicated, in degradation buffer (50 mM MES 6.5, 100 mM KCl, 10 mM MgCl_2_, and 2 mM TCEP). Degradation reactions were incubated for 30 min at 30 °C, and reactions were terminated by the addition of EDTA (50 mM). Degradation products were applied to prewashed 3 kDa Nanosep spin-filters with Omega membrane (Pall). Samples were centrifuged at 14,000 × *g* for 20 min at 23 °C, and the fluorescence of the filtrate was measured on an Agilent Eclipse fluorometer with excitation and emission wavelengths set for 490 and 525 nm, respectively.

Degradation reactions using nonlabeled MinD were monitored in reactions containing MinD (6 μM) or MinD(R3E) (6 μM), where indicated, MinC (6 μM), MinE (6 μM), where indicated, ClpX (1.0 μM), ClpP (1.2 μM), ATP (8 mM), and an ATP-regenerating system containing creatine phosphokinase (50 μg ml^−1^) and creatine phosphate (30 mM), where indicated, in degradation buffer. Immediately following the addition of ATP, and after 30, 60, 120, and 180 min, where indicated, samples were removed from degradation reactions and added to lithium dodecyl sulfate loading buffer. Protein amounts were analyzed by SDS–PAGE and densitometry.

Degradation of Gfp–ssrA (0.5 μM) was measured by monitoring loss of fluorescence over time in reactions with ClpX (1.0 μM), ClpP (1.2 μM), ATP (8 mM), and an ATP-regenerating system in degradation buffer (50 mM MES 6.5, 100 mM KCl, 10 mM MgCl_2_, and 2 mM TCEP). Fluorescence was measured at excitation and emission wavelengths of 395 and 510 nm, respectively.

### Copolymer assays with MinC and MinD

To monitor MinCD copolymer formation by light scatter, MinD (8 μM) was added to reactions containing MinC (4 μM) in copolymer assembly buffer (20 mM MES, pH 6.5, 100 mM KCl, and 10 mM MgCl_2_). A light scatter of 90° was monitored with excitation, and emission wavelengths set to 450 nm. After 5 min, ATP (8 mM) or ClpX (0.75 μM), ClpX(E185Q) (0.75 μM), ClpP (0.9 μM), or ClpX (0.75 μM), and ClpP (0.9 μM), where indicated, and ATP (8 mM) and ATP-regenerating system containing creatine phosphokinase (50 μg ml^−1^) and creatine phosphate (30 mM), were added, and 90° light scatter was monitored for an additional 30 min. In reactions monitoring copolymer destabilization, 90° light scatter was initially measured in reactions containing MinD (8 μM) or MinD(R3E) (8 μM), where indicated, MinC (4 μM), and ATP (4 mM) for 30 min at 23 °C. Then, ClpX (0.75 μM), ClpP (0.9 μM), or ClpX (0.4–0.75 μM), or ClpP (0.5–0.9 μM), where indicated, ATP (4 mM) and ATP-regenerating system containing creatine phosphokinase (50 μg ml^−1^) and creatine phosphate (30 mM) were added, and 90° light scatter was monitored for an additional 15 min.

### Electron microscopy

Reactions containing MinC (4 μM), MinD (8 μM), and MinD(R3E) (8 μM), where indicated, with ClpX (0.8 μM) and ClpP (0.9 μM), ATP (8 mM), and ATP-regenerating system containing creatine phosphokinase (50 μg ml^−1^) and creatine phosphate (30 mM) in copolymer assembly buffer. Reactions were applied to 300-mesh carbon/formvar-coated grids, fixed with glutaraldehyde (2%) and stained with uranyl acetate (2%). Samples were imaged by TEM using a JEM-2100 80 Kev instrument.

### Fluorescence microscopy

Wildtype, overexpression, and deletion strains containing *gfp–minC* and *gfp–minD* genes (Table 1), where indicated, were grown and visualized by differential interference contrast microscopy or confocal fluorescence microscopy. Oscillations in cells were observed, and rates were calculated as described previously (7). Briefly, cells were grown to log phase (absorbance of 0.3 at 600 nm) or stationary phase (16 h overnight culture), where indicated, and applied to agarose gel pads (5% w/v) containing M9 minimal medium supplemented with 0.2% glucose. Live cells were visualized with a Zeiss LSM 700 confocal microscope, and images were captured by an AxioImager M2. Oscillation rate was measured by identifying the fluorescence focal center per cell using National Institutes of Health Image J and tracking focus movement across the longitudinal cell axis (nm min^−1^) in a time series of live cell images, collected in 5 s intervals for 20 frames. Strains containing pBR322 and pClpXP were grown in the presence of ampicillin (100 μg ml^−1^) to maintain the presence of the plasmids.

### Antibiotic chase and immunoblotting

Stationary phase bacterial cultures in Luria–Bertani medium at an absorbance of 0.05 at 600 nm were grown at 30 °C for 16 h. Log phase cultures were grown overnight and diluted into fresh Luria–Bertani medium at an absorbance of 0.05 at 600 nm and grown to absorbance of 0.3 AU at 600 nm at 30 °C. Samples of 1 ml or 5 ml, respectively, were collected at 0, 30, 60, 120, and 180 min. Spectinomycin (Sigma) (200 μg ml^−1^) was added at 0 min. Proteins were precipitated with 15% trichloroacetic acid (Sigma) for 30 min at 4 °C. Suspensions were then centrifuged at 5000 × *g* for 10 min at 4 °C. Pellets were isolated and washed with acetone for 10 min at 4 °C followed by centrifugation at 10,000 × *g* for 10 min at 4 °C. Acetone was removed, and pellets were resuspended in 10% SDS. Protein samples were analyzed by reducing SDS–PAGE and transferred to a nitrocellulose membrane (Invitrogen). Membranes were washed with Tris-buffered saline (pH 7.6) and Tween-20 (0.05%), blocked for 2 h with 2% (w/v) bovine serum albumin, probed with rabbit MinD polyclonal antibody serum and goat anti-rabbit IgG coupled with horseradish peroxidase. MinD was visualized using Pierce ECL chemiluminescent Western blotting substrate (ThermoFisher, Waltham, MA), and relative levels were quantified by densitometry using ImageJ (National Institutes of Health).

## Data availability

All data pertinent to this work are contained within this article or available on request. For requests, please contact Jodi Camberg at the University of Rhode Island, cambergj@uri.edu.

## Conflict of interest

The authors declare that they have no conflicts of interest with the contents of this article.
