# Supplementary material for: Degradation of MinD oscillator complexes by Escherichia coli ClpXP
Source: J Biol Chem. 2020 Dec 10;296:100162. doi: 10.1074/jbc.RA120.013866 (PMC7857489; doi:10.1074/jbc.RA120.013866)
Supplement: Figures S1 and S2 [file mmc1.pdf]

Fig. S1

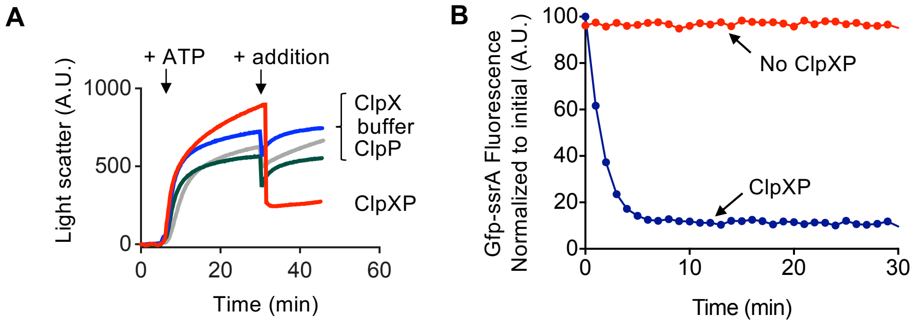

Fig. S1. MinCD copolymer stability in assembly reactions and ClpXP activity. (A) MinCD copolymers were stimulated to assemble with ATP (8 mM) and monitored by 90° light scatter in reactions containing MinC (4 µM), MinD (8 µM) and an ATP regenerating system. Where indicated ClpP (0.5 µM) (teal), ClpX (0.5 µM) (blue), individually and together (red), or an equivalent amount of buffer (gray) was added and copolymers were monitored for an additional 20 min. Each curve is representative of at least three replicates. (B) Gfp-ssrA (0.5 µM) fluorescence was measured by monitoring in the absence (red circles) and presence (blue circles) of ClpX (1.0 µM), ClpP (1.2 µM), ATP (5 mM) and a regenerating system, as described in *Experimental procedures*. Each curve is representative of at least three replicates.

Fig. S2

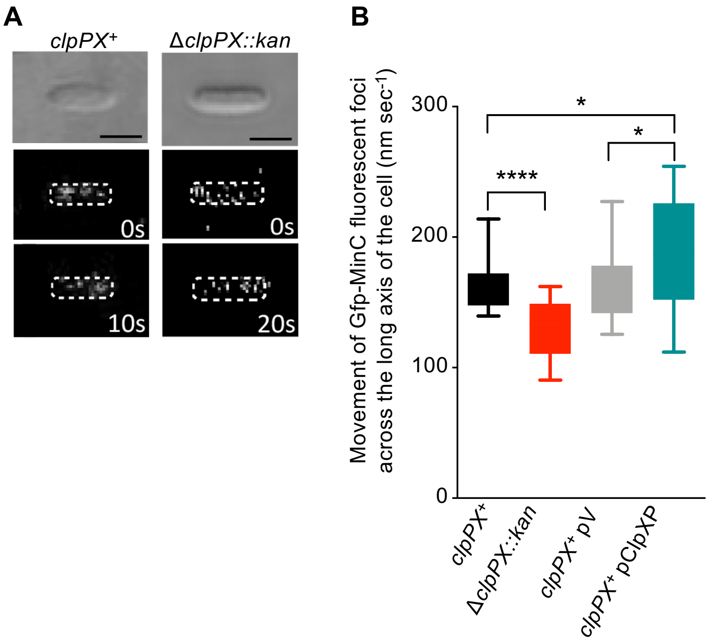

Fig. S2. Intracellular oscillation of Gfp-MinD and Gfp-MinC. (A) Cells expressing Gfp-MinD from the native locus with (CF005) and without *clpPX* (CF015) on the chromosome were visualized by fluorescence microscopy to monitor polar oscillation of Gfp-MinD of log phase cultures. Scale bar is 1 μm. (B) Oscillation rates of Gfp-MinC were measured in at least 20 cells expressing ClpXP from the chromosome (*clpPX*$^{+}$) (CL0428), deleted for *clpPX* (Δ*clpPX*::*kan*) (CF020), or overexpressing ClpXP from a vector (pClpXP) as described in *Experimental procedures* (p-v alues are as follows, '*' < 0.05, '****' < 0.0001).
